# Recurrent Zoonotic Transmission of Nipah Virus into Humans, Bangladesh, 2001–2007

**DOI:** 10.3201/eid1508.081237

**Published:** 2009-08

**Authors:** Stephen P. Luby, M. Jahangir Hossain, Emily S. Gurley, Be-Nazir Ahmed, Shakila Banu, Salah Uddin Khan, Nusrat Homaira, Paul A. Rota, Pierre E. Rollin, James A. Comer, Eben Kenah, Thomas G. Ksiazek, Mahmudur Rahman

**Affiliations:** International Centre for Diarrheal Diseases Research, Bangladesh, Dhaka, Bangladesh (S.P. Luby, M.J. Hossain, E.S. Gurley, S. Banu, S.U. Khan, N. Homaira); Institute for Epidemiology Disease Control and Research, Dhaka (B.-N. Ahmed, N. Homaira, M. Rahman); Centers for Disease Control and Prevention, Atlanta, Georgia, USA (P.A. Rota, P.E. Rollin, J.A. Comer, T.G. Ksiazek); University of Washington School of Public Health and Community Medicine, Seattle, Washington, USA (E. Kenah)

**Keywords:** Nipah virus, Bangladesh, disease transmission, respiratory tract infections, viruses, zoonoses, Pteropus, research

## Abstract

Human Nipah outbreaks recur in a specific region and time of year in Bangladesh. Fruit bats are the reservoir host for Nipah virus. We identified 23 introductions of Nipah virus into human populations in central and northwestern Bangladesh from 2001 through 2007. Ten introductions affected multiple persons (median 10). Illness onset occurred from December through May but not every year. We identified 122 cases of human Nipah infection. The mean age of case-patients was 27 years; 87 (71%) died. In 62 (51%) Nipah virus–infected patients, illness developed 5–15 days after close contact with another Nipah case-patient. Nine (7%) Nipah case-patients transmitted virus to others. Nipah case-patients who had difficulty breathing were more likely than those without respiratory difficulty to transmit Nipah (12% vs. 0%, p = 0.03). Although a small minority of infected patients transmit Nipah virus, more than half of identified cases result from person-to-person transmission. Interventions to prevent virus transmission from bats to humans and from person to person are needed.

## Abstract 2

More than half of identified cases result from person-to-person transmission.

Human Nipah virus infection, characterized primarily by fever and encephalitis, was first recognized in a large outbreak of 276 reported cases in peninsular Malaysia and Singapore that occurred from September 1998 through June 1999 (*1**,**2*). Contact with sick pigs was the primary risk factor for infection (*3*). A newly identified porcine respiratory and neurologic syndrome developed in some pigs infected with the Nipah virus; this syndrome was characterized by fever, barking cough, behavioral changes, uncoordinated gait, spasms, and myoclonus (*4*). The outbreak of Nipah virus infection in humans ceased when the infected herds of pigs in the region were culled (*5*).

Substantial data implicate fruit bats (*Pteropus* spp.) as the natural reservoir host of Nipah virus. In initial studies after the Malaysia outbreak, 16/64 (25%) *P. vampyrus* and *P. hypomelanus* fruit bats had neutralizing antibodies to Nipah virus (*6*). Nipah virus was subsequently isolated from urine specimens collected under a *P. hypomelanus* roost from partially eaten fruit dropped during feeding activity in Malaysia (*7*) and from urine collected under a *P. lylei* roost in Cambodia (*8*). Nipah virus–specific RNA was identified in saliva and urine samples from *P. lylei* fruit bats in Thailand (*9*).

The nucleotide sequences of Nipah virus strains isolated from pigs and persons in Malaysia were remarkably similar (*5**,**10**,**11*) and suggest that the entire outbreak was caused by 1 or 2 closely related strains. Indeed, all human cases of Nipah infection in Malaysia and Singapore could have originated from a single or perhaps 2 introductions of Nipah virus from its bat reservoir into pigs (*10**,**12*).

In Bangladesh, by contrast, recurrent Nipah outbreaks have been recognized since 2001 (*13**–**17*), and the strains of Nipah isolates show substantial heterogeneity in their nucleotide sequences (*11*). This heterogeneity suggests repeated introductions of Nipah virus from its host reservoir into the human population in Bangladesh.

A single species of fruit bats of the genus *Pteropus*, *P. giganteus*, lives in Bangladesh and is widely distributed throughout the country (*18*). Blood samples from *P. giganteus* bats in Bangladesh and neighboring India commonly have antibodies to Nipah virus (*13**,**19*). The conditions that permit recurrent introduction of Nipah virus from fruit bats to persons in Bangladesh are unknown. Besides the tendency for Nipah virus outbreaks to reoccur in Bangladesh, a second notable difference in Nipah virus epidemiology in Bangladesh is that, in contrast to Malaysia, where person-to-person transmission of Nipah virus was not confirmed (*20*), person-to-person transmission has been repeatedly observed in Bangladesh (*15**,**16*).

The high prevalence of antibodies to Nipah virus among *Pteropus* spp. bats suggests that Nipah virus is well adapted to transmission between individual bats of that genus. We hypothesize that when a *Pteropus* spp. bat sheds Nipah virus in Bangladesh, this virus occasionally infects 1 or more persons. Once people are infected, the epidemic chain of transmission can be perpetuated by person-to-person transmission (*16*).

A more complete understanding of the character of Nipah virus infection in Bangladesh has been limited by the analysis of relatively small individual outbreaks. We combined data from the 7 recognized human outbreaks and the identified sporadic cases of Nipah virus in Bangladesh from 2001 through 2007. The objective was to describe the introduction of Nipah virus into the human population and the epidemiology of person-to-person transmission.

## Methods

We reviewed available data from investigations of all of the human Nipah infections recognized in Bangladesh from 2001 through 2007. Information from the separate investigations was combined into a single database. Not all variables of interest were collected from the earliest outbreaks, but because many of the same investigators were involved across the outbreaks, data were collected in similar formats.

Persons were classified as being infected with Nipah virus if they had fever with new onset of altered mental status, seizures, or severe shortness of breath and either had specific antibodies against Nipah virus or were part of a cluster of similar case-patients in the same region, with at least 1 of the case-patients being Nipah-antibody positive. In addition, if a person had fever and immunoglobulin (Ig) M antibody to Nipah, that person was classified as being infected with Nipah virus.

We classified Nipah cases as part of a cluster if at least 1 other Nipah case was identified in the same community within 3 weeks of onset of illness. If no other cases appeared in the same community within 3 weeks, the Nipah case was classified as an isolated case.

We counted distinct introductions of Nipah virus into the human population. Each cluster of Nipah case-patients and each sporadic case was counted as a separate Nipah introduction.

We classified persons as primary case-patients if illness developed without known contact with any other Nipah case-patients, as secondary case-patients if Nipah disease developed 5–15 days after close contact with other Nipah case-patients, and as Nipah spreaders if at least 1 person with whom that person had close contact had Nipah illness develop 5–15 days after that contact. We collected geographic coordinates by using global positioning systems from the home of each case-patient.

### Laboratory Methods

The field team, which involved a large number of different people over the course of the many outbreak investigations, centrifuged whole blood specimens and brought the separated serum on wet ice to the laboratory at the International Center for Diarrheal Diseases Research, Bangladesh (ICDDR,B), where it was stored at –70°C. Before 2007, serum samples were shipped on dry ice to the US Centers for Disease Control and Prevention (CDC) and tested with an IgM capture enzyme immunoassay (EIA), which detects Nipah IgM, and with an indirect EIA for Nipah IgG (*21*). Nipah (Malaysia prototype) virus antigen was used in both assays. In 2007, the Nipah antibody testing was conducted at the government of Bangladesh’s Institute of Epidemiology Disease Control and Research using reagents provided by the Special Pathogens Branch of CDC in Atlanta. All positive samples were confirmed at CDC.

### Statistics

We assessed whether differences in proportions were more extreme than would be expected by chance by using the χ^2^ test or the Fisher exact test when the expected cell size was <5. The basic reproductive number (R_0_) is the average number of persons infected by an infectious person during his or her entire infectious period when he/she enters a totally susceptible population (*22*). We estimated R_0_ by dividing the number of persons infected by a primary case-patient by the total number of primary case-patients.

### Ethics

Many of these data were collected as part of routine surveillance or emergency outbreak investigations, so study protocols did not undergo human subjects review. In 2005, a protocol for establishing a formal Nipah surveillance system and the strategy for outbreak investigations were reviewed and approved by the Ethical Review Committee of the ICDDR,B.

## Results

A total of 122 Nipah case-patients were identified; 74 (61%) were male, and the mean of all case-patients age was 27 years (range 2–75). Eighty-seven (71%) of 122 died. Fifty-nine (48%) infections were serologically confirmed. Of the 63 case-patients that were not laboratory confirmed, 59 (94%) died before any blood was collected; 4 (6%) had an initial serum specimen without detectable antibodies but died before a follow-up specimen could be collected.

**Figure 1:**
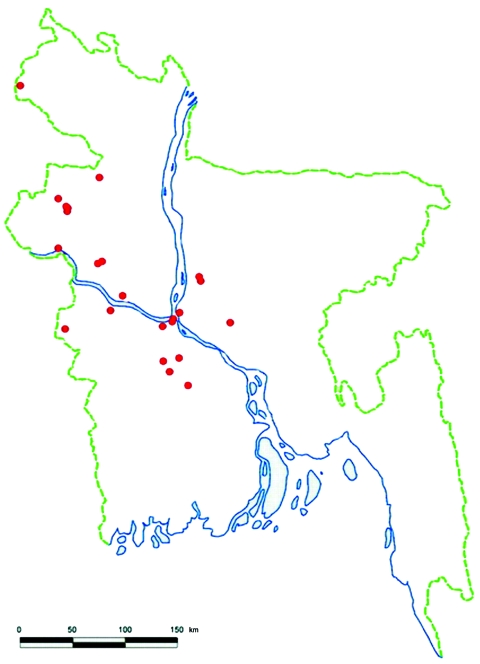
Locations of human Nipah virus introductions (red dots), Bangladesh, 2001–2007.

We identified 23 introductions of Nipah virus into human populations in central and northwestern Bangladesh from 2001 through 2007 (Figure 1). Ten introductions involved clusters with a median of 10 affected persons (range 2–35). Thirteen Nipah introductions resulted in isolated human infections.

**Figure 2:**
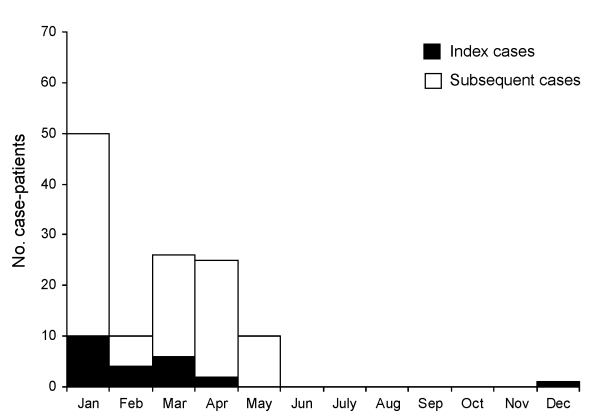
Human Nipah virus infections in Bangladesh, by month of illness onset, 2001–2007.

**Figure 3:**
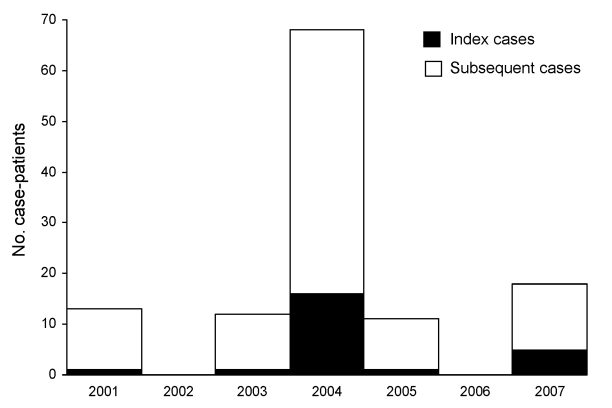
Human Nipah virus infections in Bangladesh, by year of illness onset, 2001–2007.

The onset of illness for the index cases for the 23 human Nipah introductions occurred from December 31 through April 20 (Figure 2). The probability that all 23 index cases would randomly occur in 5 contiguous months is (5 / 12)^23^ × 12 = 0.00000002. The number of Nipah case-patients varied by year; most cases were recognized in 2004, and no cases were recognized in 2002 and 2006 (Figure 3).

In 62 (51%) Nipah case-patients, illness developed from apparent person-to-person transmission. None of these cases were professional healthcare workers exposed to Nipah case-patients on the job. The case-fatality ratio was similar for primary and secondary cases (75% vs. 66%, respectively; p = 0.28).

Nine case-patients (7%) were Nipah spreaders who transmitted Nipah infection to other persons. All Nipah spreaders died; 4 of these were classified as secondary cases. Nipah case-patients who had difficulty breathing during their illness were more likely to be Nipah spreaders (12% vs. 0%, p = 0.03). Similarly, Nipah case-patients who had cough were more likely to be Nipah spreaders (12% vs. 2%, p = 0.080). Patients classified as secondary cases were just as likely to have difficulty breathing as those classified as primary cases (65% vs. 66%, p = 0.94).

Nipah case-patients who died were more likely to be Nipah spreaders than were Nipah case-patients who survived (10% vs. 0%; Fisher exact test, p = 0.057). Persons who spread Nipah were no more likely to be primary Nipah case-patients than were Nipah patients who did not spread Nipah (56% vs. 49%, odds ratio 1.3, 95% confidence interval 0.34–5.1, p = 0.74). Nipah spreaders transmitted Nipah to a mean of 7 persons (range 1–22).

Among the 10 clusters, 5 involved person-to-person transmission ranging from 2 to 5 generations of transmission. The 60 primary Nipah case-patients infected 29 subsequent persons. The estimated basic reproductive number was 0.48.

Fourteen secondary Nipah case-patients had contact with a primary Nipah case-patient for <2 days. The median incubation period of these secondary cases was 9 days (range 6–11 days).

## Discussion

These introductions of Nipah virus from its presumed reservoir in *Pteropus* bats to humans in Bangladesh were clustered, both in time of year as well as in specific years. Occurring during winter and spring in certain years, this clustering in Bangladesh suggests that the specific conditions necessary for Nipah virus transmission from bats to humans occurs only periodically. Perhaps shedding of Nipah virus by *Pteropus* bats is seasonal, and because of population dynamics and the accumulation of susceptible juvenile bats over time, transmission is quite low in some years compared with other years when widespread shedding and transmission occur.

The presumed wildlife reservoir of Nipah virus, bats of the genus *Pteropus*, is widely distributed across Bangladesh, the rest of the Indian subcontinent, and Southeast Asia (*18*). Wherever *Pteropus* bats have been collected and tested, they commonly have had antibodies against Nipah or a related virus (*8**,**9**,**13**,**23**,**24*). Human Nipah infections in Bangladesh, however, have been recognized in a confined geographic area. The Institute of Epidemiology Disease Control and Research in Bangladesh has national surveillance for disease outbreaks. Outbreaks involving the deaths of several previously healthy persons, which is characteristic of Nipah outbreaks, typically generate substantial local concern, media attention, and notification of local and central health authorities. Despite investigations of outbreaks in all regions of the country, all confirmed Nipah outbreaks have occurred in the same central and northwestern regions (Figure 1). Notably, the only 2 outbreaks that have been reported from India have been in regions within 50 kilometers of the border with Bangladesh and immediately contiguous with the affected areas in Bangladesh (*25**,**26*). One hypothesis that would explain this geographic concentration of human cases is that the bats are attracted to specific foods available in these areas during the winter and spring; people living in these communities are occasionally exposed to foods contaminated with bat urine or saliva that contains Nipah virus. One outbreak of Nipah virus was associated with consumption of raw date palm sap, which is harvested from December through March in this region (*14*). In 21 of the 23 recognized index case-patients, Nipah virus illness developed during this harvest season.

Person-to-person transmission is a major pathway for human Nipah virus infection in Bangladesh, accounting for 51% of recognized cases in this review. By contrast, in Malaysia and Singapore, person-to-person transmission was not confirmed, although 1 nurse who cared for Nipah patients in an intensive care unit in Malaysia and reported no clinical illness had serologic evidence of Nipah virus infection and brain magnetic resonance imaging consistent with Nipah virus infection (*27*). Even if occasional unrecognized person-to-person transmission of Nipah virus occurred in the outbreak in Malaysia, person-to-person transmission is much more apparent and common in Bangladesh. Moreover, the outbreak in Siliguri, India, in 2001 was also characterized by widespread person-to-person transmission (*25*).

Three factors likely contributed to the higher frequency of person-to-person transmission of Nipah virus in Bangladesh than was observed in Malaysia. First, respiratory disease associated with Nipah infection was more common and more severe in Bangladesh compared with that in Malaysia and Singapore (*28**–**30*). Earlier studies demonstrated that Nipah virus was present in respiratory secretions of some Nipah virus–infected patients (*11**,**31*). We found that Nipah case-patients who had difficulty breathing were much more likely to be Nipah spreaders. Together, these findings suggest that when a Nipah virus–infected patient has a symptomatic respiratory tract infection associated with Nipah virus, the patient can shed an infectious inoculum of Nipah virus in his respiratory secretions. In the largest recognized Nipah outbreak in Bangladesh, touching a patient who had respiratory difficulties was a risk factor for developing Nipah infection (*16*). The personal care typically provided to ill and dying relatives in Bangladesh is likely a second important contributor to person-to-person transmission. This care is characterized by close physical interaction, frequent contact with a patient’s saliva, and a lack of basic infection control practices because the paradigm of communicable disease is inconsistent with the prevailing cultural interpretation of illness (*32*). Third, all Nipah virus strains from human cases in Malaysia were genetically similar, in contrast to the marked diversity of the strains in Bangladesh (*11*). Some strains possibly possess genetic characteristics that facilitate person-to-person transmission.

Our 0.48 estimate of the basic reproductive number of Nipah virus in rural Bangladesh, a resource-poor setting with extremely limited infection control practices, suggests that Nipah virus is unlikely to cause a sustained human pandemic from person-to-person transmission. This conclusion is further supported by our observation of 23 separate introductions of the virus into the human population; none of these introductions resulted in sustained person-to-person transmission. Indeed, only 1 of the introductions exceeded 2 generations of transmission (*16*). However, we could study only 9 patients who transmitted the Nipah virus. Thus, our understanding of the variability in transmission of different strains of the virus in different contexts is limited.

This analysis has limitations. First, we almost certainly did not identify all human Nipah virus infections that occurred in Bangladesh from 2001 through 2007. Many persons in Bangladesh, especially the impoverished, do not access the formal healthcare system, even when seriously ill (*33**,**34*). The available data on human Nipah virus infection in Bangladesh are biased toward infections acquired in outbreaks recognized by public health authorities. Because meningoencephalitis is a common cause of hospitalization in Bangladesh, sporadic cases that are unrecognized as Nipah virus infection may be the more common presentation of Nipah virus infection in the country. Indeed, during 2004 and 2007, years when multiple outbreaks were identified, many patients who sought treatment for symptoms of encephalitis in hospitals located near identified outbreak areas were tested for Nipah virus. This testing identified several additional patients infected with the virus who lived quite a distance from the outbreak villages and who had no apparent connection to other cases. Thus, surveillance almost certainly underestimates the public health impact of Nipah virus infection in Bangladesh and may underestimate its geographic and seasonal range. Because outbreaks are more likely to lead to recognition of Nipah virus infection than sporadic cases and person-to-person transmission can occur only in clusters, the overall proportion of Nipah virus infections in Bangledesh that are transmitted person to person is probably <50%.

A second limitation is that the calculation of the basic reproductive rate assumed that all persons infected by a primary case-patient were identified. The investigation team could have failed to recognize all cases, especially in persons with milder or asymptomatic infection. In Malaysia, among 178 persons without symptoms who lived on farms where at least 1 person with confirmed Nipah encephalitis was identified, 20 (11%) had antibodies against Nipah virus (*3*). This possibility of asymptomatic infection suggests that our estimate of the basic reproductive rate is a minimal estimate. However, in outbreaks, when mild or asymptomatic persons were screened in Bangladesh, few additional Nipah cases were identified (J. Hossain, pers. comm.). Moreover, only patients who died transmitted the infection, so the possible infection of persons in whom mild illness developed is unlikely to contribute to the risk for pandemic transmission.

A third limitation is that we identified only 9 persons who transmitted Nipah virus and so have limited statistical power to assess their characteristics. Indeed, the association of cough with Nipah virus transmission and death with Nipah transmission are above the traditional guideline for statistical significance. However, the weight of the evidence, including an association with respiratory illness that meets the traditional criterion for statistical significance and the isolation of Nipah virus in respiratory secretions, suggests that person-to-person transmission occurs occasionally from virus-infected patients who are efficient respiratory transmitters of the virus.

A fourth limitation is that this analysis assumes that persons in whom Nipah illness developed 5–15 days after contact with a Nipah patient were considered infected by the contact. If the subsequent case-patient had a similar environmental exposure to Nipah virus as the initial case-patient, this approach may overestimate the proportion of cases that result from person-to-person transmission. However, Nipah virus is readily recovered from the saliva of infected persons (*11**,**31*), and epidemiologic studies of individual outbreaks implicate contact with Nipah-infected patients as a risk factor for transmission (*16*). Moreover, the observed 6–11-day window between exposure and disease is consistent with incubation periods for other human viral infections. Thus, person-to-person transmission is the most likely route of transmission in these scenarios.

The recurrent introduction of Nipah virus from its fruit bat reservoir to humans in Bangladesh causes outbreaks with high fatality rates and substantial neurologic sequelae among survivors (*35*). Fruit bats have a critical niche in the ecosystem, contributing to both plant pollination and distribution of seeds (*36**,**37*). Preventing human Nipah virus infections in Bangladesh is difficult because these infections are relatively rare compared with many other serious and more common health threats faced by the large population of this low-income country. Improved understanding of the mechanism of zoonotic transmission from bats to humans may help identify feasible approaches to prevent future introductions of the virus into the human population. Interventions to reduce the risk for pathogen exposure by minimizing saliva contact or improving hand hygiene among persons who care for seriously ill patients in severely resource-constrained settings could reduce person-to-person transmission of Nipah as well as transmission of other dangerous pathogens. In confirmed or highly suspected cases of Nipah virus infection, respiratory droplet precautions may also be warranted.
